# Atomistic understanding of cation exchange in PbS nanocrystals using simulations with pseudoligands

**DOI:** 10.1038/ncomms11503

**Published:** 2016-05-10

**Authors:** Zhaochuan Fan, Li-Chiang Lin, Wim Buijs, Thijs J. H. Vlugt, Marijn A. van Huis

**Affiliations:** 1 Process and Energy Department, Delft University of Technology, Leeghwaterstraat 39, 2628CB Delft, The Netherlands; 2 Soft Condensed Matter, Debye Institute for Nanomaterials Science, Utrecht University, Princetonplein 5, 3584CC Utrecht, The Netherlands

## Abstract

Cation exchange is a powerful tool for the synthesis of nanostructures such as core–shell nanocrystals, however, the underlying mechanism is poorly understood. Interactions of cations with ligands and solvent molecules are systematically ignored in simulations. Here, we introduce the concept of pseudoligands to incorporate cation-ligand-solvent interactions in molecular dynamics. This leads to excellent agreement with experimental data on cation exchange of PbS nanocrystals, whereby Pb ions are partially replaced by Cd ions from solution. The temperature and the ligand-type control the exchange rate and equilibrium composition of cations in the nanocrystal. Our simulations reveal that Pb ions are kicked out by exchanged Cd interstitials and migrate through interstitial sites, aided by local relaxations at core–shell interfaces and point defects. We also predict that high-pressure conditions facilitate strongly enhanced cation exchange reactions at elevated temperatures. Our approach is easily extendable to other semiconductor compounds and to other families of nanocrystals.

## Abstract (web summary)

Cation exchange is a promising technique to modify ionic nanostructures by replacing the existing cations with those provided by the solution. Here, the authors use molecular dynamics to study cation exchange in PbS nanocrystals by combining solvent and ligand effects into a pseudoligand parameter.

## Graphical abstract

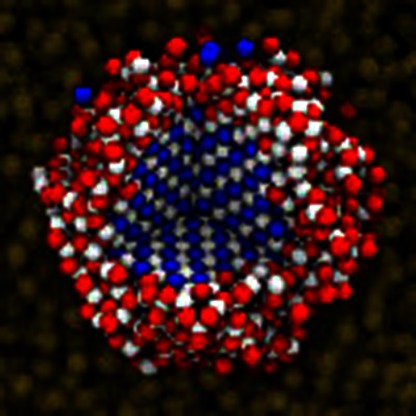

To synthesize novel nanocrystals (NCs) and heteronanocrystals (HNCs) with designed shape and structure, cation exchange (CE) has recently been extensively explored and applied^1^,^2^,^3^,^4^,^5^,^6^,^7^,^8^,^9^,^10^,^11^. This process involves colloidal ionic NCs whereby the cations in the NCs are (partially) replaced by the cations from the surrounding solution. The CE technique has greatly boosted the syntheses of nanostructures with various morphologies. Starting from elementary NCs that are synthesized using well-known synthesis routes such as hot injection^12^, the CE technique can be used to modify nanostructures into other novel nanostructures or heterostructures such as binary rods^13^ and core/shell structures^10^,^14^. The CE technique can be extremely powerful when combined with other advanced (post)syntheses techniques such as oriented attachment and seeded growth, which leads to many novel nanostructures including nanooctapods^8^, nanorod couples^15^ and 2D honeycomb superlattices^16^.

Pb→Cd exchange (that is, replacing Pb^2+^ by Cd^2+^) in lead chalcogenide NCs is a typical CE process that produces PbE/CdE (E=S, Se, Te) core/shell HNCs^14^,^17^,^18^. This CE process for colloidal PbS NCs can be expressed as follows:


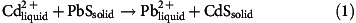


A typical experimental route of this CE consists of two steps^14^,^19^: (1) synthesis of the PbS parent NCs, and (2) immersing the parent NCs in Cd-oleate solutions and applying heat for 0.5–10 h at 373–473 K. PbS has a rocksalt (RS) crystal structure where the atoms have a sixfold coordination, whereas CdS has a zinc blende (ZB) crystal structure where the atoms have a fourfold coordination. The lattice mismatch between these two structures is <2% (ref. 20). Pb→Cd CE in PbE NCs has often been described as a self-limiting process given that a core/shell structure is generally found in the product. Complete conversion is usually difficult to achieve^18^,^19^,^21^. Compared to other types of CE^4^,^22^ (for example, Cd→Ag) that take place spontaneously at ambient temperature and at a shorter timescale, Pb→Cd CE requires a relatively high temperature and a long time to overcome a large energy barrier^1^.

Although the Pb→Cd CE process has been studied by modern experimental techniques such as energy dispersive X-ray spectrometry, high-resolution transmission electron microscopy (HRTEM) and 3D electron tomography^17^,^19^,^21^,^23^,^24^, a detailed understanding of the atomistic mechanism is still missing. Theoretical investigation of this CE process using molecular simulations also remains a great challenge. Understanding the thermodynamics and kinetics of CE at the atomic scale is crucial for the future development of the precise control of this technique. In this study, classical molecular dynamics (MD) simulations are used to study Pb→Cd CE in PbS NCs within a solution. Our results indicate that the rate of CE can be controlled by changing the ligand type, or by adjusting the temperature and/or pressure. By changing the ligand type, the direction of CE can also be controlled, which enables the reverse CE process to take place. Furthermore, we reveal the atomistic mechanism of ion transportation in the NCs. During CE, Pb ions are kicked out by exchanged Cd interstitials and migrate through interstitial sites. Local structural distortions and point defects present at the PbS/CdS interface and in the CdS shell region assist the Pb jumps by locally expanding the voids in Cd sublattice and by reducing the Coulombic repulsion between Pb and Cd ions.

## Results

### Thermodynamic driving force

**Figure 1:**
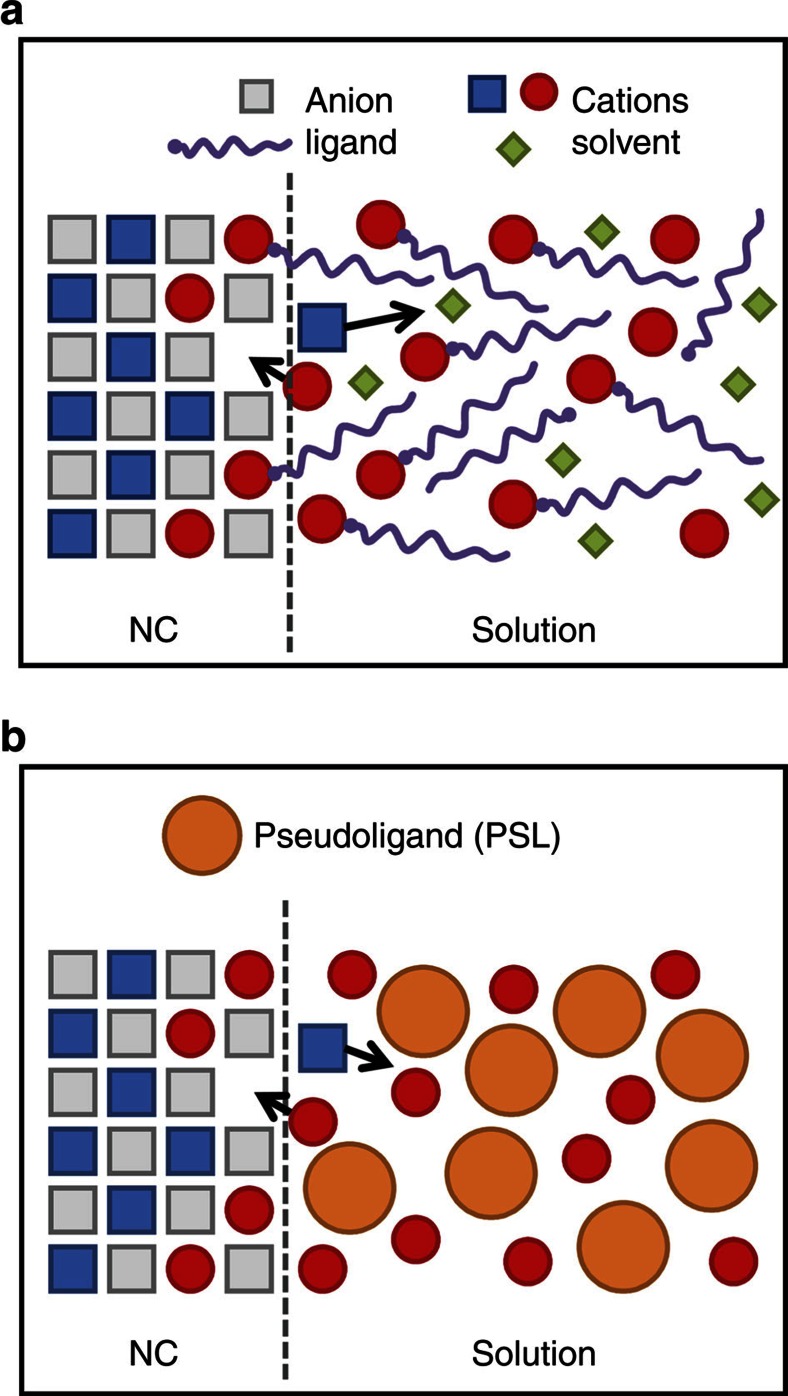
Schematic representations of a CE process. (**a**) CE in experiments. (**b**) CE in MD simulations with a coarse-grained PSL model. The dashed lines indicate the NC/solution boundaries. The black arrows indicate the motion of the cations during CE.

As schematically shown in Fig. 1a, CE not only involves parent NCs, but also the surrounding solution, consisting of exchanging cations, ligands and solvent molecules. CE is initiated at the interface between the NCs and the solution and then proceeds to occur within the NCs. Modelling the whole system atomistically requires large system sizes and long simulation times, which is not computationally feasible. To overcome this, we developed a coarse-grained model to mimic the cation–ligand solution in which the effects of ligands and solvent are incorporated into negatively charged large spherical particles (Fig. 1b, the orange circles). These particles are denoted by pseudoligands (PSLs). PbS–CdS systems are modelled using our newly developed all-atom force field^20^, which accurately describes the crystal structures, elastic properties, polymorphic stabilities, and surface energies of PbS and CdS solids and Pb_*x*_Cd_1−*x*_S mixed phases. Details about the coarse-grained PSL model and the all-atom force field are provided in Methods section, Supplementary Methods, Supplementary Discussion, Supplementary Figs 1,2 and Supplementary Table 1.

**Figure 2:**
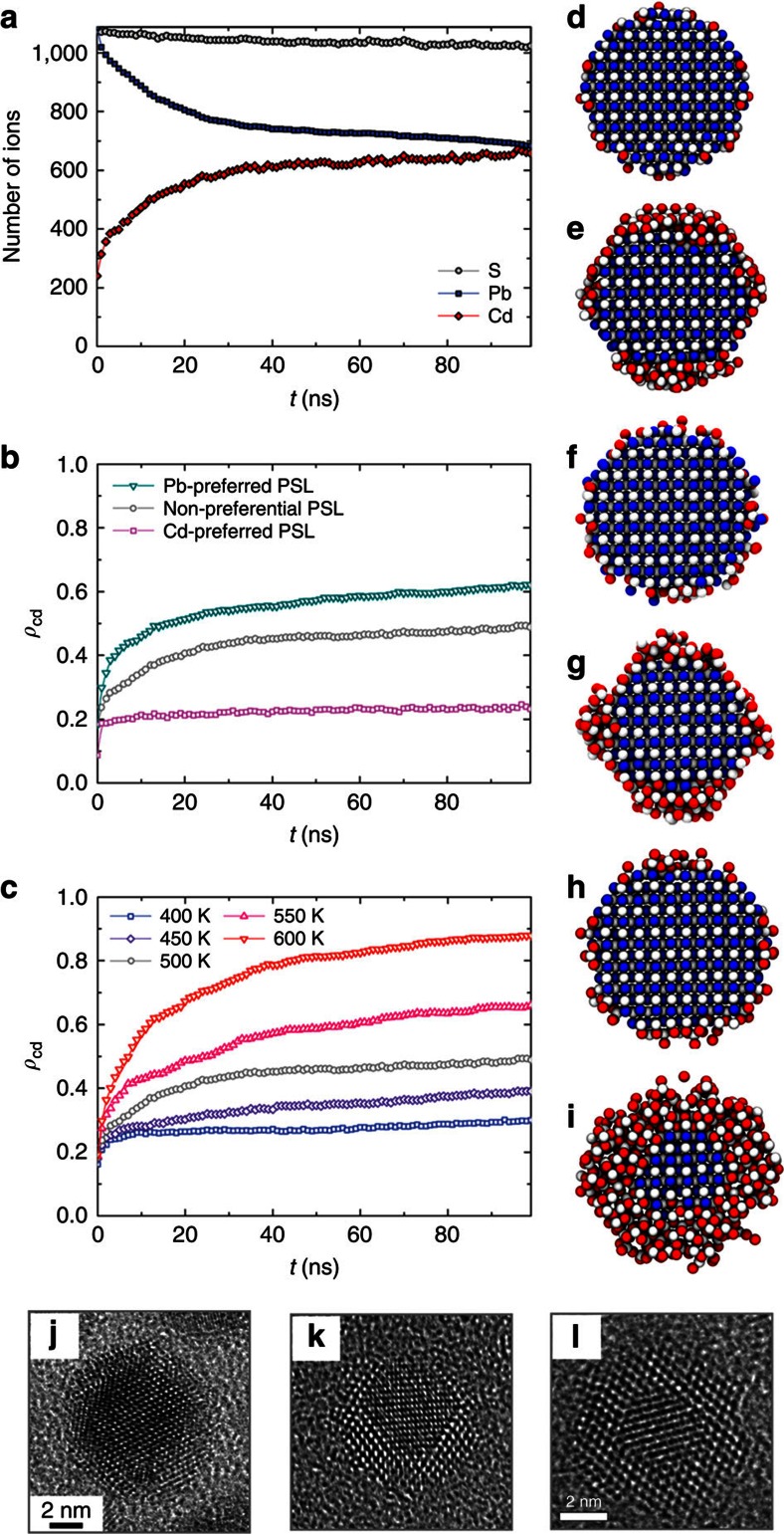
The Pb→Cd CE process in a 4.7-nm-sized PbS NC using different PSLs at different temperatures. (**a**) Number of in-going Cd ions (red diamonds), remaining Pb ions (blue squares) and S ions (grey circles) in the parent PbS NCs as a function of time. MD simulations were performed at 500 K with non-preferential PSLs. (**b**,**c**) Time evolution of exchange ratio *ρ*_Cd_ (the ratio of in-going Cd ions over the total number of cations in the NC). (**b**) Simulations performed at 500 K with different PSLs. A constant value of *ρ*_Cd_ (∼0.2) for simulation with Cd-preferred PSLs indicates that CE did not take place. The low fraction of Cd ions corresponds to those Cd ions adsorbed at the NC surface. (**c**) Simulation performed using non-preferential PSLs at different temperatures. (**d**–**i**) Snapshots of the RS-(110) sections of the NCs in 100-ns MD simulations: (**d**) initial configuration; (**e**–**i**) final configurations of 100-ns simulations at 500 K with non-preferential PSLs (**e**), Cd-preferred PSLs (**f**), and Pb-preferred PSLs (**g**) and those with non-preferential PSLs at 400 K (**h**) and 600 K (**i**). The white, blue and red spheres represent S, Pb and Cd, respectively. (**j**–**l**) HRTEM images of PbE/CdE core/shell HNCs achieved by experimental CE, in a [110] projection. (**j**) A 9-nm-sized PbS/CdS core/shell HNC, image adapted with permission from (ref. 25) (copyright 2014 American Chemical Society). (**k**) A 5.6-nm-sized PbSe/CdSe core/shell HNC, image adapted with permission from (ref. 26) (copyright The Royal Society of Chemistry 2011). (**l**) A 6.6-nm-sized PbTe/CdTe core/shell HNC, image adapted with permission from (ref. 17) (copyright 2009 American Chemical Society).

The thermodynamic driving force for Pb→Cd CE is controlled by two main factors: the free energy difference between the two materials forming the NCs and the solubility of both cations in the ligand solution^1^,^2^. The former depends on several factors, such as the lattice energy, surface energy, size and shape of the NCs, the interfacial energy when forming HNCs and the entropy of mixing. The dominating factor is the lattice energy difference between ZB–CdS and RS–PbS, as the difference in surface energies and the lattice mismatch between ZB–CdS and RS–PbS are relatively small^20^. During CE, the change in *T*Δ*S* is typically one or two orders of magnitude smaller than that from the enthalpy change (Δ*H*) at ∼500 K (ref. 24), and thus the entropy of mixing is also not a dominating factor^21^. The lattice energy of ZB–CdS computed using our force field is about 0.3 eV per formula unit (eV/f.u.) less than that of RS–PbS^20^. Therefore, if the PSL–Cd and PSL–Pb interatomic interactions are equivalent (that is, using non-preferential PSLs, see Methods section) and when the temperature is sufficiently high, the relatively low-lattice energy of ZB–CdS will promote the Pb→Cd CE process in PbS NCs. MD simulations of a 4.7-nm-sized PbS nanocrystal immersed in a non-preferential PSL solution were carried out at 500 K. Pb→Cd CE was indeed observed in our simulations. Figure 2a shows that, as a function of simulation time, the number of Pb cations in the parent NC decreases, whereas the number of Cd cations increases, clearly demonstrating the exchange of cations. We also observed that the number of S anions remained nearly constant, which indicates that, as the anionic sublattice is more stable, the anionic sublattice is preserved during the CE process. The CE process found in the MD simulations is in excellent agreement with experimental observations^21^: the exchanging Cd ions in the solutions initially cover the surface of the parent NC (that is, corresponding to the sharp increase of the number of in-going Cd ions during the first few nanoseconds as shown in Fig. 2a), while the surface Pb ions in the parent NC are dissolved into the solution (Supplementary Movies 1 and 2). Subsequently, the Cd ions diffuse inwards into the NCs. When the Cd ions occupy the sites of the first few outermost monolayers (MLs), the exchange slows down, forming a PbS/CdS core/shell HNC (see Fig. 2e; Supplementary Movies 1 and 2). The formation of the CdS shell impedes the outward diffusion of the Pb ions from the PbS core. As the exchange slows down, the number of in-going Cd 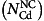, the number of remaining Pb in the NC 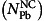 and the exchange ratio (*ρ*_Cd_), defined as the number of in-going Cd ions divided by the total number of cations in the NC (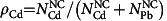, grey circles in Fig. 2b,c), become nearly constant after a few tens of nanoseconds. The PbS/CdS core/shell HNCs with higher exchange ratios (for example, the final configuration of an exchanged HNC at 600 K in a MD simulation of 100 ns in Fig. 2i) have a structure and morphology, which is very similar to those of experimentally observed core/shell HNCs, as shown in HRTEM images of PbS/CdS (Fig. 2j), PbSe/CdSe (Fig. 2k) and PbTe/CdTe (Fig. 2l) configurations^17^,^25^,^26^.

The preferential dissolution of the cations in solutions with different ligands has a crucial role in determining the direction and exchange ratio of CE. Using the same system and temperature (that is, the 4.7-nm-sized PbS NC immersed in a Cd–PSL solution at 500 K), we investigated the influence of different ligand solutions on the CE process by considering different PSL–cation interactions. For Pb-preferred, non-preferential and Cd-preferred PSL solutions (see Methods section), the exchange ratio *ρ*_Cd_ as a function of simulation time is shown in Fig. 2b. As shown in Fig. 2b, Pb→Cd CE can be significantly accelerated while a Pb-preferred solution is used, and it can also be completely prohibited using a Cd-preferred solution. Note that a small amount of Cd (*ρ*_Cd_∼20%) was found in the NC with the Cd-preferred PSLs. These Cd ions did not penetrate into the NC but were adsorbed at the NC surface (Fig. 2f). We also found that, by immersing a ZB–CdS NC in a solution containing Pb ions and Cd-preferred PSLs, the system underwent a reverse Cd→Pb CE (see Supplementary Methods and Supplementary Fig. 3). The reverse CE did not take place in a non-preferential or a Pb-preferred PSL solution. As shown experimentally, a way to achieve both the forward and backward Pb↔Cd CE is to change the type of ligand: Cd-oleates are often chosen as cation–ligands solutions to achieve Pb→Cd CE^14^,^19^,^21^, whereas aqueous Pb nitrate solutions, PbCl_2_–oleylamine and Pb–oleates–oleylamine have been used to achieve the reverse CE process^27^,^28^,^29^,^30^.

The temperature is another key factor for determining the equilibrium and the exchange rate of CE. MD simulations for PbS nanocrystals immersed in non-preferential PSL solutions were performed at temperatures ranging from 400 to 600 K. The CE process was found to be faster at higher temperatures with a more complete Pb→Cd replacement (Fig. 2c). At 600 K, nearly 90% of the cations in the NC was replaced by Cd within 100 ns, and a CdS shell with a thickness of about four MLs was formed (Fig. 2c,i). In experiments, high temperatures often lead to severe damage and distortion of the NCs (among others, Ostwald ripening can take place)^19^,^21^. In the simulations, we also observed that at 700 K the whole NC dissolved in the solution. Note that temperatures used in the MD simulations should not be directly compared with those in experiments. The timescale in our MD simulations is generally of the order of hundreds of nanoseconds, which is much shorter than a typical timescale of the Pb→Cd CE experiments (mins–hr)^14^,^17^,^18^,^19^,^21^. Accordingly, a higher temperature is required in our simulations in order to accelerate the CE process and so that it falls within the timescale accessible by MD.

### Self-limiting exchange

**Figure 3:**
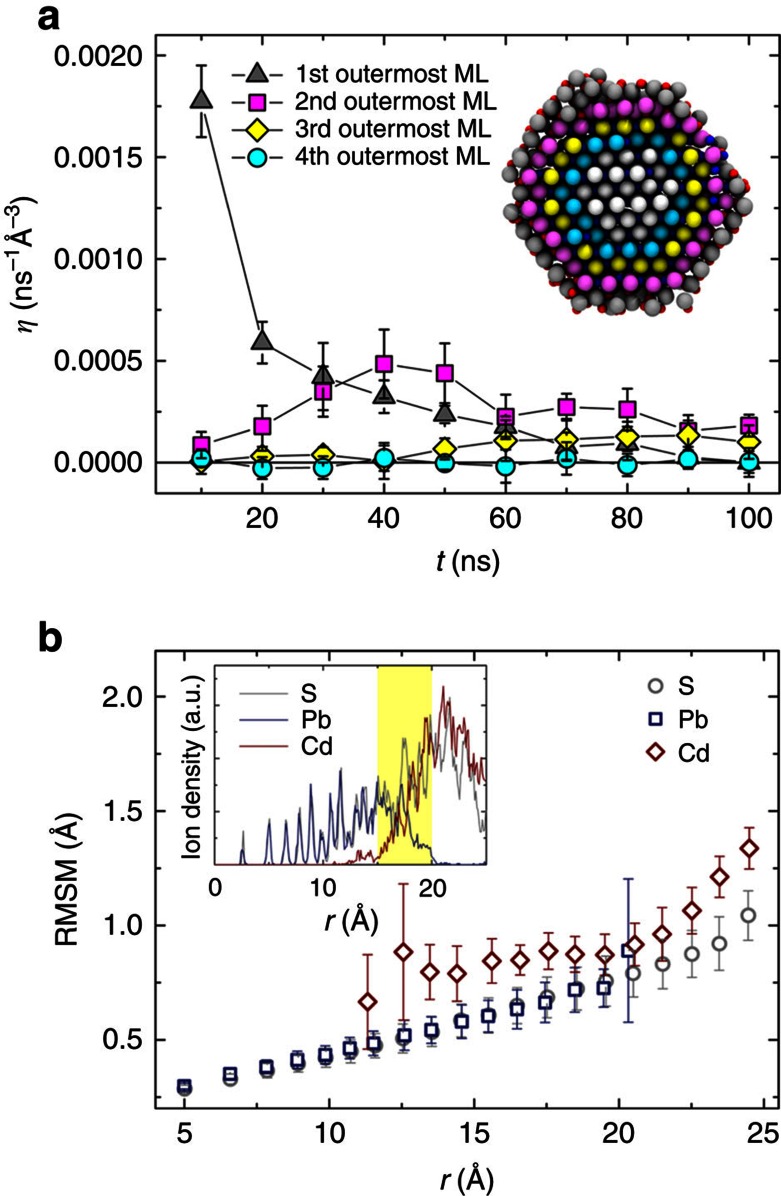
Exchange rate and atomic mobility analysis of Pb→Cd CE. (**a**) Time evolution of the averaged volume-scaled exchange rate *η* (see Methods section) for the four outermost atomic MLs. Data were collected from 10 independent 100-ns MD simulations of a PbS NC at 550 K. The inset shows the final configuration of the RS-(110) section from one of the MD simulations. The S ions are coloured according to the colour code of the different outermost MLs. The small red and blue spheres are Cd and Pb, respectively. (**b**) Averaged RMSM (see Methods section) of S (grey circles), Pb (blue squares) and Cd (red diamonds) as a function of *r* (*r* is the distance between the ion and the centre of the NC). The inset shows the ion density as a function of *r*. The yellow region labels the Cd_*x*_Pb_1−*x*_S mixed phase domain between the PbS core and the CdS shell. Data were sampled during the last 100 ps of the ten independent 100-ns MD simulations.

One of the appealing features of CE at the nanoscale is the fast exchange rate compared to the process at the macroscopic scale. Nonetheless, Pb→Cd CE is often reported to be self-limiting. Although complete Pb→Cd exchange has been experimentally reported^16^,^18^,^21^, accurate controls, high temperatures and long times are required. Figure 3a shows the time evolution of the averaged volume-scaled exchange rate *η* (see Methods section) at the first four outermost MLs of the PbS NC from the MD simulations at 550 K with non-preferential PSLs. The CE process takes place in a layer-by-layer fashion. The exchange front moves from the first outermost ML to the second ML in 20∼40 ns and further proceeds to the third outermost ML after ca. 50 ns onward (Fig. 3a; Supplementary Movies 1 and 2; Supplementary Fig. 4). The exchange rate of the fourth layer is always zero, suggesting that the exchange front has not yet reached the fourth ML in 100 ns. The overall exchange rate *η* significantly decreases as the exchange front moves inward. This is consistent with the average root mean square motion (RMSM, see Methods) calculated for different ionic species as a function of the distance from the initial position of an ion to the centre of the NC (denoted by *r*) as shown in Fig. 3b. To clarify the ionic positions in the PbS/CdS core/shell structure, the radial densities of each ionic species are plotted in the inset of Fig. 3b. The RMSM of all types of ions increases as the ions are closer to the NC surface, indicating a faster CE process at the surface than inside the NC. Obviously, the large surface-to-volume ratio of nanostructures leads to a swift CE process at the nanoscale, but the spherical morphology of the PbS parent NCs and the layer-by-layer mode of exchange in the Pb→Cd CE process determine its self-limiting nature. This has also been reported experimentally for an analogous Cd→Zn CE process in CdSe NCs^10^.

It is instructive to point out that the radial density of each atom type (the inset of Fig. 3b) indicates a pure PbS domain (*r*≤15 Å), a Cd_*x*_Pb_1–*x*_S mixed phase domain (15 Å<*r*<20 Å) and a pure CdS domain (20 Å≤*r*<24 Å). Considering the inhomogeneity in PbS/CdS HNCs, the mixed phase layer with a thickness of 5 Å is relatively thin. It has been also shown experimentally that PbS/CdS HNCs obtained by CE have a sharp PbS/CdS boundary^17^,^19^. Unlike systems containing materials with the same crystal structure, which easily form mixed phases or solid solutions (for example, the CdSe/ZnSe^10^ and CdSe/CdS/ZnS^31^ systems), PbS and CdS in HNCs undergo a phase separation even at relatively high temperatures^24^,^26^.

### Kinetic mechanism

**Figure 4:**
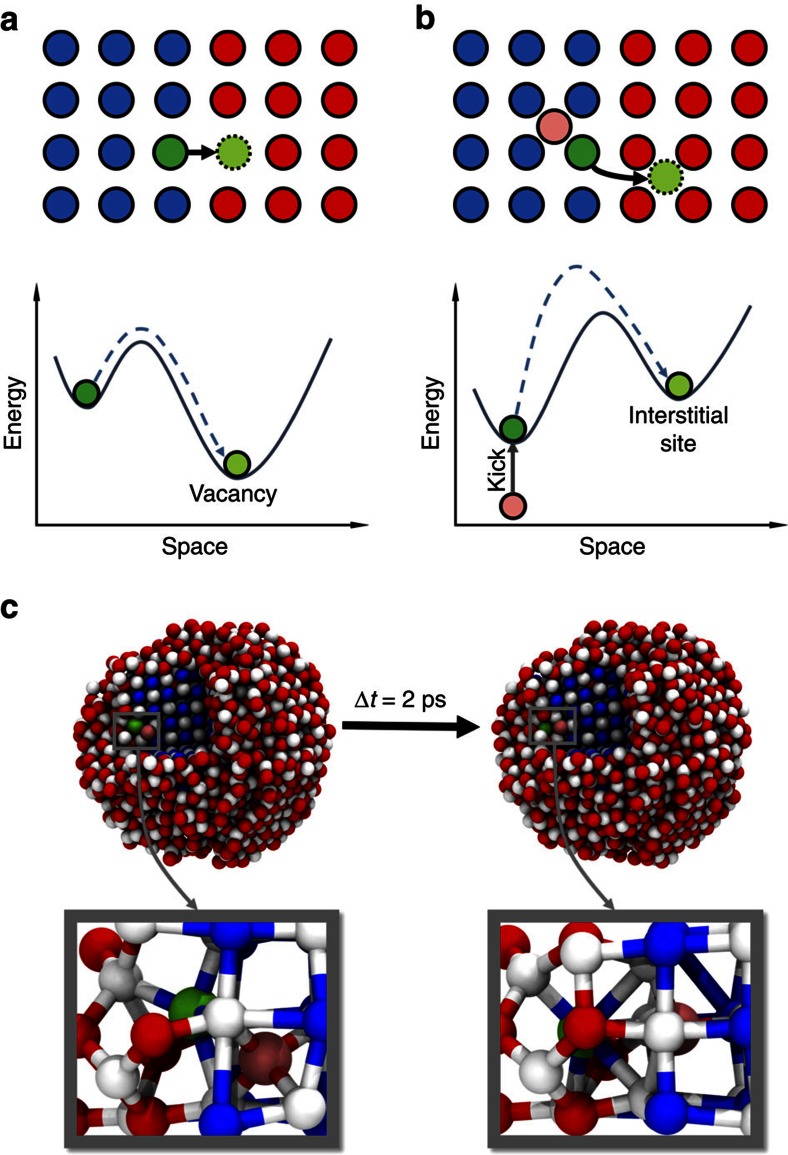
‘Vacancy-mediated' mechanism versus ‘kick-out' mechanism. (**a**,**b**) Schematic representations of the ‘vacancy-mediated' mechanism (**a**) and the ‘kick-out' mechanism (**b**). The blue and red circles represent Pb and Cd, respectively. The green circles denote the Pb ions migrating at the PbS/CdS interface. The pink circle is a Cd interstitial kicking the Pb ion out of the PbS core region. The lower panels of (**a**,**b**) schematically show the energy landscapes of these two different mechanisms. (**c**) Typical snapshots from MD simulations showing the ‘kick-out' mechanism. Left and right parts of (**c**) are the configurations of the NC before and after the jump of a Pb ion (the green sphere) out of the PbS core, respectively. The Pb jump is initiated by a Cd interstitial (the pink sphere) in the PbS region. The blue, red and white spheres are Pb, Cd and S, respectively. The lower panels of (**c**) are magnifications of the region where the ‘kick-out' motion takes place.

Based on a variety of experimental data, two mechanisms have been proposed for describing the kinetics of CE: a ‘vacancy-mediated' mechanism^18^,^21^,^24^ and a ‘kick-out' mechanism^32^. The ‘vacancy-mediated' mechanism suggests that Pb ions from the PbS core migrate through Cd vacancies^18^,^21^,^24^ (Fig. 4a). These Cd vacancies form at the surface of NCs and migrate further towards the PbE/CdE interface, transporting the Pb ions through the CdS shell into the solution. In a recent paper, Bothe *et al*.^32^ challenged the ‘vacancy-mediated' mechanism by pointing out that the formation energy of Cd vacancies is rather high, and that therefore, cations migration through vacancies is unlikely. These authors proposed that the Pb ions are kicked out of the PbS core by exchanged Cd ions (the ‘kick-out' mechanism) and migrate trough interstitial sites^32^ (Fig. 4b). From an energetic point of view, the ‘vacancy-mediated' mechanism suggests that the Pb jumps in the CE process are assisted by ‘energy traps' (vacancies) and thermal fluctuations (Fig. 4a), whereas the ‘kick-out' mechanism suggests that the Pb jumps are stimulated by Coulombic repulsion between the Pb ions and exchanged Cd interstitials (Fig. 4b). Due to the limitations in resolution of state-of-the-art experimental techniques, it is difficult to determine which mechanism is correct for the Pb→Cd CE process. By analysing the atomic trajectories in our simulations, we are able to unveil the mechanism for CE. We divide the CE process into two steps: (1) CE at the PbS/CdS interface and (2) the migration in the CdS shell region. We frequently observed the ‘kick-out' mechanism^32^ at the PbS/CdS interface in our simulations. Figure 4c shows a typical ‘kick-out' motion: two MD snapshots showing configurations before and after the jump of a (green coloured) Pb ion out of the PbS core. An exchanged Cd ion (the pink sphere) located at an interstitial site in the PbS core region is present in the pre-jump configuration. The exchanged Cd interstitial kicks the Pb ion out of the PbS core by Coulombic repulsion and then occupies its site. We identified many more Pb jumps at the PbS/CdS interfaces from five independent 200 ns MD simulations on which we performed statistical analysis (see Supplementary Methods and Supplementary Table 2). Exchanged Cd interstitials near the jumping Pb ions can be found in about 47% of the identified Pb jumps, strongly supporting the ‘kick-out' mechanism.

**Figure 5:**
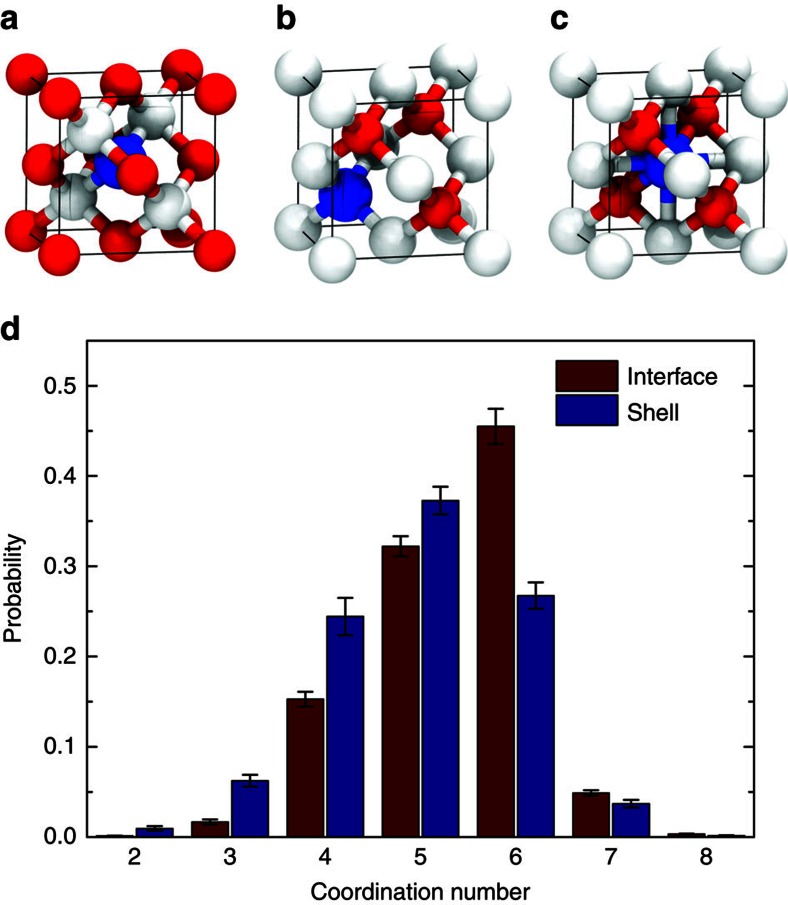
Statistical analysis of different Pb jumps in the Pb→Cd CE. (**a**–**c**) Three different sites for a Pb ion in ZB–CdS: (**a**) a tetrahedrally coordinated interstitial site; (**b**) a tetrahedrally coordinated substitutional site; (**c**) an octahedrally coordinated interstitial site. The blue, red and white spheres are Pb, Cd and S, respectively. (**d**) Distribution of the coordination numbers of the jumping Pb ions after the jumps at the PbS/CdS interface and in the CdS shell region, based on an analysis of about 10,000 Pb jumps identified from five independent 200-ns MD simulations.

Another difference between the ‘vacancy-mediated' and ‘kick-out' mechanisms is whether the Pb ions migrate through vacancies or interstitial sites during CE. We examined the pre-jump atomistic environment (that is, the distribution of anions and cations) around the post-jump locations of the jumping Pb ions (see Supplementary Methods). If a Pb ion jumps into a ZB–CdS domain, it is mostly located at one of the two different cation sites: the tetrahedral cation site (Fig. 5a,b) or the octahedral cation site (Fig. 5c). The former is a tetrahedrally coordinated site with four nearest neighbour anions whereby the Pb ion can be either located at an interstitial (Fig. 5a) or at a substitutional site (Fig. 5b). The octahedrally coordinated site has six nearest neighbour anions so that the Pb ion can only be an interstitial (Fig. 5c). By counting the coordination numbers of the nearest neighbour anions around the positions in which the Pb ions jumped, the tetrahedral and octahedral cation sites can be distinguished (see Supplementary Methods). Figure 5d shows the coordination numbers for the identified Pb jumps in the interfacial and shell regions. The results indicate that at the PbS/CdS interface, the Pb ions show a strong preference to jump to octahedral cation sites (coordination number 6) rather than to tetrahedral cation sites (coordination number 4). A slight preference for the octahedral cation sites can be found when the Pb ions are migrating in the CdS shell region. We calculated the averaged distances between the four nearest anions and the jumping Pb ions 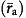, and those between the four nearest cations and the jumping Pb ions 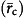 in the post-jump configurations (see Supplementary Table 3). The distance analysis included the Pb jumps at the PbS/CdS interface and in the CdS shell region with anionic coordination numbers 4 and 6. Note that coordination numbers 3, 5 and 7 correspond to local structural distortions or off-site Pb ions, which were excluded from the distance calculation. The results show that the values of 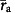 are in a narrow range of 3.0–3.1 Å, regardless of the tetrahedral/octahedral site type and the region (interface or shell) where the Pb jumps took place. The values of 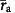 are consistent with the Pb–S bond length in the bulk crystal PbS (3.0 Å), indicating that the locations of the Pb ions in the CdS domains are mainly determined by Pb–S interactions. For the Pb ions located at the tetrahedral cation sites, the ratio of 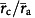 is about 1.13 (see Supplementary Table 3), which suggests that these Pb ions are indeed interstitials (the value of 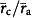 for a tetrahedrally coordinated Pb interstitial is 1.15) rather than substitutional ions (the value of 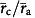 for a substitutional Pb is 1.63). For the Pb ions located at the octahedral cations sites, 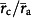 is about 1.08 (see Supplementary Table 3). This value is significantly larger than the value of ideal Pb interstitials at octahedral cation sites (0.87). The mismatch can only be explained by local structural distortions and the formation of Cd vacancies. The statistical analysis of our simulations supports the ‘kick-out' mechanism^32^ whereby Pb ions migrate through interstitial sites and not through vacancies during CE. However, the migration of the Pb ions through octahedral sites needs to be assisted by Cd vacancies.

## Discussion

The exchange rate in our simulations is faster than that in experiments, and this Pb→Cd CE reaction rate may be attributed to the higher reaction temperature and the higher concentrations of Cd in the solution compared to those in experiments. In Pb→Cd CE experiments of PbS NCs, the reaction temperature is typically in the range of 100–200 °C (373–473 K). In our simulations, the highest temperature is 600 K in order to accelerate the CE process, considering that the timescale of our simulations is several orders of magnitude shorter than of experiments. Such a high temperature can increase the probability of cation jumps by two to six orders of magnitude. Raising the temperature in the simulations bears the risk that the kinetic mechanism found in our simulations may differ from that in experiments at a lower temperature. However, as the atomistic configurations of the exchanged nanocrystals in our simulations are very similar to those observed in experiments (Fig. 2e–l), it is anticipated that the mechanisms are the same. The coarse-grained PSL model was used to mimic the ligand and solvent molecules. The size of a PSL is smaller than that of actual ligands used in CE experiments, and the number of the guest cations (Cd) is set equal to the number of PSLs. Therefore, the concentration of guest cations in the solution is higher in our simulations in comparison to experiments (Supplementary Discussion), which may also result in a faster exchange in the simulations. It should be noted that our simulations are only able to show the initial stages (the first few hundreds of nanoseconds) of the CE process. Compared to the exchanged NCs in experiments that has formed CdS shells with a thickness of 1–2 nm (Fig. 2j–l), the exchanged NC at 400 K (within the temperature range used in CE experiments) only has formed an incomplete and thin layer of CdS (thickness of the shell <0.3 nm) after 100 ns (Fig. 2h), thus the CE took place to a lesser extent. To show a CE process for this NC in a simulation at 400 K with a similar degree of exchange as those in experiments, the required simulation time may be extremely long (>1 s), which is not computationally feasible.

Increasing the temperature is an effective way to accelerate the CE process, however, it has been reported that Ostwald ripening takes place by heating colloidal PbS NCs at 473 K, thus destroying the monodispersity of the NCs^19^. A multiple step heating scheme^18^,^19^ has been therefore used for CE experiments at high temperatures in which the NCs are protected by CdS shells that form at a lower temperature. We did find significant dissolution of S ions in our simulations at high temperatures (Supplementary Movies 1 and 2; Supplementary Fig. 5), however, Ostwald ripening could not be directly observed in our simulations since a single NC was considered. The dissolved S anions may also recrystallize on other parts of the same NC with the cations in the solution, thus enabling massive migration of anions and reconstruction of PbE/CdE HNCs. This phenomenon becomes more important at high temperatures and/or with active ligands, in some extreme cases PbE NCs or PbE/CdE core/shell HNCs may transform to a Janus-like heterostructure^26^,^29^,^33^.

Besides the multiple step heating scheme, we found that increasing the pressure could be an alternative approach to preserve NCs at elevated temperatures in order to accelerate the CE processes and to achieve complete compositional conversions. The Pb→Cd CE process cannot be accelerated by merely applying a hydrostatic pressure to the solution (Supplementary Methods and Supplementary Fig. 6), however, an increased hydrostatic pressure is able to increase the melting point of ionic semiconductor solids^34^ and their stability at high temperatures. For example, only five Pb ions remained in the exchanged NC (*ρ*_Cd_=0.997) in a MD simulation of 100 ns at 750 K and a hydrostatic pressure of 1.00 GPa, and a PbS NC completely transformed into a CdS NC in 70 ns at 800 K and 1.0 GPa (Supplementary Fig. 6). At a standard pressure the NC would have been severely damaged upon reaching a temperature of 700 K, whereas the exchanged NC remains solid phase and retains a quasi-spherical morphology at high-temperature–high-pressure conditions (*p*=1.00 GPa, *T*≤800 K, Supplementary Fig. 6). Our simulations therefore suggest that a high-pressure–high-temperature approach is a promising method to control the rate and degree of the Pb→Cd CE process.

The exchange process is initiated by Cd interstitials in the PbS domain, whereby these Cd interstitials kick Pb ions out of the PbS core. A high temperature is needed to promote the penetration of these guest cations into the NC, and this explains why some types of CE processes (for example, Pb→Cd and Zn→Cd) only take place upon annealing. Although it has been shown that Pb ions in Pb→Cd CE migrate through interstitial sites, it is important to note that Cd vacancies, or most likely, Cd Frenkel pairs (a Cd ion moves to a neighbouring interstitial site, thereby forming a vacancy and an interstitial) play a role during the exchange process, especially at the PbS/CdS interface. Since PbS has a sixfold RS structure, there should be a strong preference for Pb ions to occupy octahedral cation sites. However, in CdS domains, Pb ions are prevented from jumping into octahedral interstitial sites due to strong Coulombic repulsion between the Pb ions and the densely packed Cd ions around the octahedral interstitial sites. Only when assisted by Cd vacancies, which are present due to the formation of Cd Frenkel pairs, Pb ions can jump into octahedral cation sites. It is well known that structural instability will be introduced by dipole moments at the polar/non-polar interface or the polar/polar interface with different crystal structures^35^. Therefore, formation of Cd Frenkel pairs and local structural distortion occurs more easily and more frequently at the PbS/CdS interface than in the CdS domain at high temperatures. This conclusion agrees with the results from our MD simulations showing that the Pb ions show a stronger preference for octahedral cation sites while jumping at the PbS/CdS interface. In general, the CE processes take place more easily and faster in the systems forming interfaces of two different structures (for example, PbE/CdE^14^,^17^,^18^, Ag_2_E/CdE^22^ and Cu_2_E/CdE^36^) than those having the same structure (for example, CdE/ZnE^10^,^31^). In addition, we point out here that the ‘vacancy-mediated' mechanism wrongly guessed the detailed kinetics of this particular CE process. Cd vacancies do not migrate back and forth between the NC surface and the PbS/CdS interface, and Pb ions do not migrate through Cd vacancies. The formation of Cd vacancies (without Cd Frenkel pairs) is indeed difficult^32^, especially at high temperatures whereby vacancies tends to be annealed out^36^. Pb substitutions in the CdS shell region have been rarely observed in our simulations.

As CdS and PbS have different crystal structures, a structural transition must take place during a CE process. Two most stable phases of CdS, wurtzite (WZ) and ZB, have almost the same cohesive energy (the difference between the cohesive energies of these two CdS polymorphs is about 1 meV/f.u. according to density functional theory (DFT) calculations^20^). During the CE process of Pb and Cd chalcogenides nanostructures, however, the RS-to-ZB transition has been more frequently observed experimentally than the RS-to-WZ transition^16^,^17^,^18^,^23^,^24^ (see Fig. 2j–l). The latter requires an extra step of anionic sublattice slips^37^ as the former only requires slips of the cationic sublattice^20^. Therefore, the RS-to-ZB transition has a lower energy barrier than the RS-to-WZ transition. In our simulations, almost equal amounts of the WZ and ZB domains were found in the CdS shell of an exchanged HNC (Supplementary Fig. 7). This discrepancy between experiments and simulations may be attributed to the adopted force field for CdS as the stability of ZB phase is slightly underestimated^20^. However, we note that the competition between the RS-to-ZB and RS-to-WZ transitions in Pb→Cd CE also depends on many other factors, including the morphology of initial parent NCs, types of ligand and solvent, temperatures and nucleation energies. For instance, the WZ stacking faults and even direct RS-to-WZ transition have been observed in CE experiments^16^,^17^,^18^,^23^,^24^. In our simulations, most of the Cd ions were located at the tetrahedral cation sites (ZB or WZ), and only those at or near the PbS/CdS interfaces may jump between tetrahedral and octahedral sites. It has been reported that CdS shells may remain in a metastable RS phase during CE processes^25^, which has not been observed in our simulations.

In summary, we used classical MD simulations to investigate Pb→Cd CE in colloidal PbS NCs. The cation-ligands solutions were simulated using a coarse-grained PSL model, allowing us to study this large system at a long-time scale. The main driving force of the Pb→Cd CE process is the competition between the ionic crystal lattice energies and the cations solvent solubility. By adjusting the PSL–cation interatomic interactions (analogous to changing the cation–ligand solutions in experiments) and the temperature, the direction and rate of the CE process can be controlled. Our simulation results reveal that the CE process is initiated by ‘kick-out' motions and that Pb ions migrate through interstitial sites. Highly mobile Cd ions at the PbS/CdS interface facilitate the formation of Cd Frenkel pairs, which enables the jumps of cations and accelerates the CE process. Our study provides a profound insight into the physics underlying the Pb→Cd CE at the atomic level. The hybrid all-atom/coarse-graining approach is able to effectively reveal both the thermodynamics and kinetics of the Pb→Cd CE, and, more importantly, this model may also be adapted to investigate other types of CE (for example, Cd→Cu and Zn→Ag) and other synthesis techniques (for example, hot injection, seeded growth and vapour–liquid–solid growth). Finally, our simulations also predict that high-temperature–high-pressure conditions can greatly enhance the CE rate. To date, several key questions remain unclear, such as: Why does CE behave very differently in different materials? What is the optimal ligand solution, temperature and pressure to yield complete Pb→Cd CE? How can the thickness and uniformity of the CdE shell be completely controlled? Our approach provides opportunities to further address these questions, and we anticipate that the coarse-grained PSL model will be extended and further improved.

## Methods

### Colloidal PbS NC systems and MD simulations

A spherical PbS NC with a diameter of 4.7 nm containing 1,088 {Pb–S} pairs was cut from a RS–PbS matrix with a lattice parameter of 6.0 Å. The PbS NC was placed in the centre of a cubic simulation box with a size of 10 nm. The simulation box was then randomly filled with 4,352 {Cd-PSL} pairs (to avoid overlaps, we ensured that the distance between any two ions is larger than 3.5 Å). Periodic boundary conditions were applied in all directions.

In MD simulations, the PSL–PSL or PSL–cation interatomic interactions consist of two parts: long-ranged Coulombic interactions and short-ranged interactions described by a Buckingham potential. We assume that electron transfer does not play an important role during Pb→Cd CE, that is, the charges of the cations, anions and anionic ligands in the solid and liquid phases, and at the solid/liquid interface, do not change. The main function of our PSL model is to create a solution in which the guest cations are dissociated and CE takes place at the NC/solution interface (see Supplementary Discussion). The parameters in the Buckingham potentials can be altered to create Pb-preferred, Cd-preferred or non-preferential PSLs (Pb/Cd-preferred means that the Pb/Cd–PSL interactions are stronger than the Cd/Pb–PSL interactions; non-preferential means that the Cd–PSL and Pb–PSL interactions are equivalent, see Supplementary Discussion, Supplementary Table 1 and Supplementary Fig. 1), so that the influence of the cation-ligand interactions on CE can be quantitatively investigated.

All MD simulations were carried out using the LAMMPS code^38^ (http://lammps.sandia.gov). The particle–particle–particle–mesh method^39^ was used to calculate the Coulombic interactions and a cut-off radius of 10 Å was set for all short-ranged interactions. The equations of motion were integrated using the velocity–Verlet algorithm with a time step of 1 fs. Four S ions in the centre of the NC were constrained at fixed positions to prevent net motion and rotation of the NCs. Simulations of a few hundreds ns were carried out in the NPT ensemble and the first 100 ps was used for preliminary equilibration at the desired temperatures and zero pressure. The longest simulation time was 300 ns.

### Volume-scaled exchange rate

Several MLs were defined in a PbS NC (one ML has a thickness of 3.5 Å, see the inset of Fig. 3a). The exchange rate in the *j*^th^ ML and at time *t*, *η*_*j*_(*t*), was defined as:


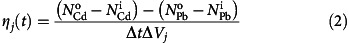


where *N* is the number of Cd or Pb ions passing through the surface o or i during a time Δ*t*. The superscripts o and i represent the outer and the inner surfaces of the *j*^th^ ML, respectively. A positive value of *N* indicates the cations are moving inward to a given ML, while a negative value indicates that the cations are moving outward. Δ*t* is a small time interval (Δ*t*=1 ps) and Δ*V*_*j*_ is the volume of the *j*^th^ ML. Using this definition, a positive value of *η* indicates a Pb→Cd CE process and a negative value indicates the reverse Cd→Pb CE. We sampled the *η*_*j*_ every 100 ps for the first four outermost MLs from simulations for a PbS NC with non-preferential PSLs at 550 K. Each data point in Fig. 3a corresponds to an averaged *η*_*j*_ over a time interval of 10 ns from 10 independent MD simulations.

### Root mean square motion

The RMSM was used to describe the vibration and mobility of ions. The RMSM was defined as:


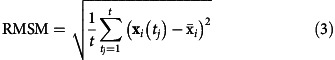


where *t* is the time to sample the data, which is the last 100 ps of 10 independent 100- ns simulations. **x**_*i*_(*t*_*j*_) is the position of ion *i* at time *t*_*j*_, 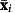 is the time-averaged position of the ion *i*. The time interval between two samplings is 1 ps. The RMSM of each ion and its initial distance *r* to the centre of the NC were calculated. The RMSM was further averaged over the same types of ions with similar values of *r* (±0.5 Å).

## Additional information

**How to cite this article:** Fan, Z. *et al*. Atomistic understanding of cation exchange in PbS nanocrystals using simulations with pseudoligands. *Nat. Commun.* 7:11503 doi: 10.1038/ncomms11503 (2016).

## Supplementary Material

Supplementary Information Supplementary Figures 1-7, Supplementary Tables 1-3, Supplementary Discussion, Supplementary Methods and Supplementary References

Supplementary Movie 1 Pb-for-Cd cation exchange taking place in a PbS NC - view along [100] direction. This movie shows the Pb-for-Cd CE of the 4.7-nm-sized PbS NC in the non-preferential PSL solution at 550 K. A (100) section of the NC is shown. The white, blue, red, and yellow spheres represent S, Pb, Cd, and PSLs respectively. The length of the movie corresponds to a simulation time of 100 ns.

Supplementary Movie 2 Pb-for-Cd cation exchange taking place in a PbS NC - view along [110] direction. This movie shows the same simulation as in Movie 1 but now displaying a (110) section of the NC.
