# Supplementary material for: Atomistic understanding of cation exchange in PbS nanocrystals using simulations with pseudoligands
Source: Nat Commun. 2016 May 10;7:11503. doi: 10.1038/ncomms11503 (PMC4866395; doi:10.1038/ncomms11503)
Supplement: Supplementary Information — Supplementary Figures 1-7, Supplementary Tables 1-3, Supplementary Discussion, Supplementary Methods and Supplementary References [file ncomms11503-s1.pdf]

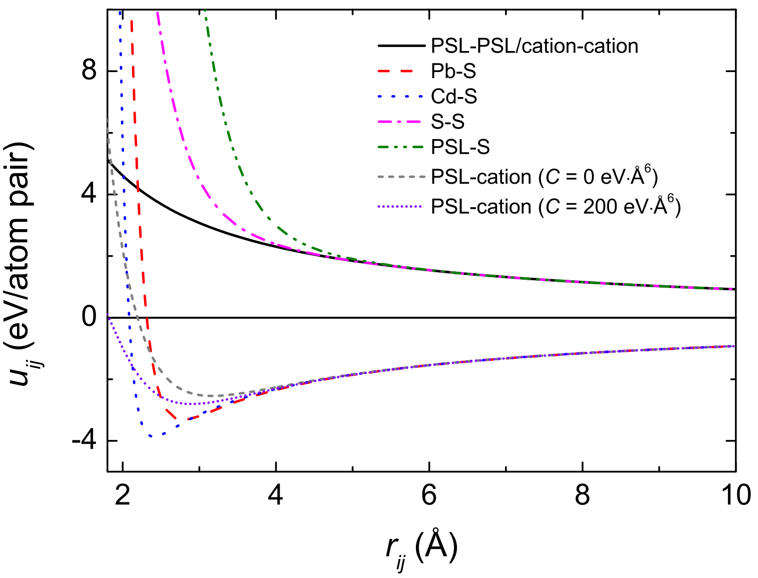

Supplementary Figure 1: **Interatomic pair potentials as a function of the interatomic distance r_{ij}.** The parameter C in the PSL-cation interatomic potentials is either 0 or 200 eV·Å^6.

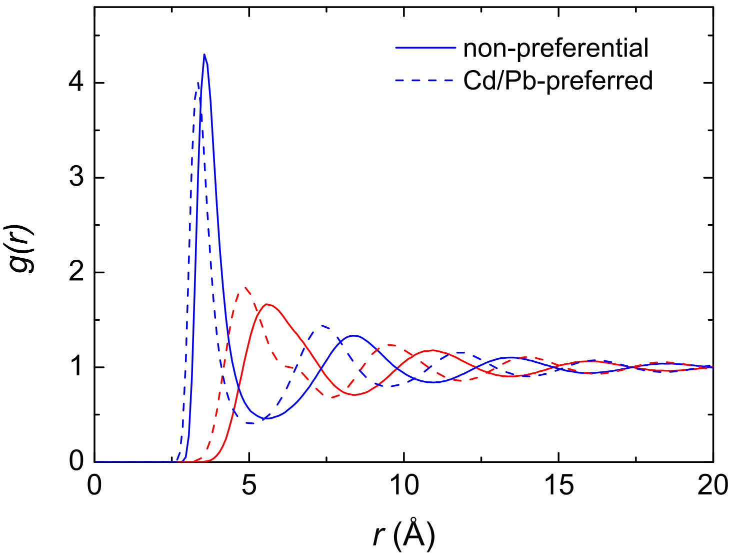

Supplementary Figure 2: **Radial distribution functions, $g(r)$, of cation/PSL mixtures at 500 K.** The mixtures consist of 864 cation-PSL pairs without a NC. The type of cations is either Cd or Pb, and the PSLs are either non-preferential or Cd/Pb preferred. MD simulations were performed for four different cation/PSL mixtures at 500 K and 0 GPa for 2 ns, in which the first 1 ns was used for equilibration. Since the cation-cation and PSL-PSL interactions are identical (equivalent Coulombic repulsion), their radial distribution functions are identical. Similarly, the radial distribution functions for mixtures with the same type of PSLs but different cations (Cd or Pb) are also indistinguishable. The blue and red lines show $g(r)$ for cation-PSL and PSL-PSL (or cation-cation), respectively. The solid and dashed curves show $g(r)$ for cation/PSL mixtures with non-preferential PSLs and Cd/Pb-preferred PSLs, respectively.

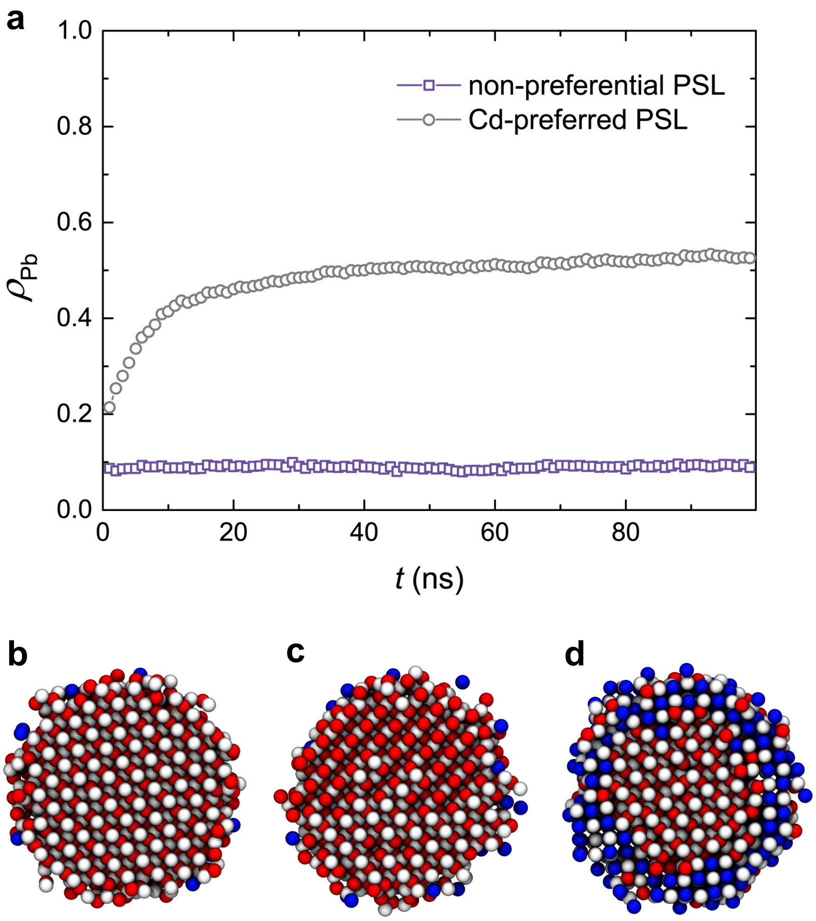

Supplementary Figure 3: **Reverse Cd→Pb CE in a ZB-CdS NC. a** Time evolution of the exchange ratio ρ_{Pb} (the ratio of in-going Pb ions over the total number of cations in the NC). Simulations were performed at 550 K with non-preferential (grey circles) and Cd-preferred (purple squares) PSLs. The constant value of $\rho_{\mathrm{Pb}} \sim 0.1$ for the simulation with non-preferential PSLs indicates that CE did not take place (see also the final configuration in **c**). **b-d** Initial (**b**) and final (**c-d**) configurations of the ZB-CdS NC for 100 ns simulations at 550 K with non-preferential (**c**) and Cd-preferred PSLs (**d**). The white, blue, and red spheres represent S, Pb, and Cd ions, respectively.

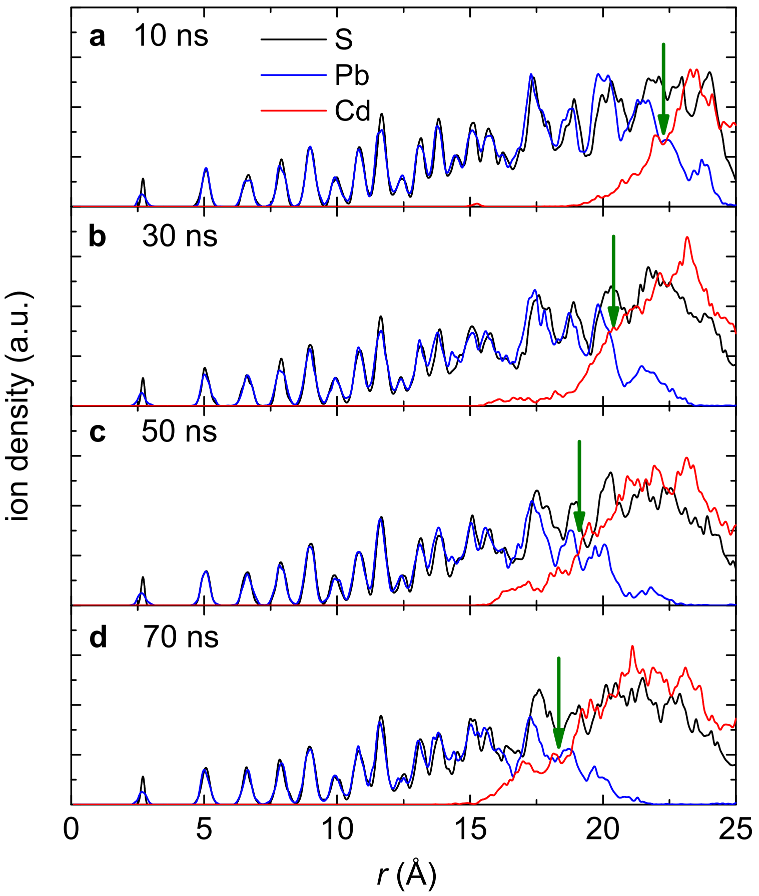

Supplementary Figure 4: **Ion density as a function of the distance from the NC centre (r) for different simulation times. a-d** MD simulations were performed for a colloidal PbS NC for 100 ns at 550 K with non-preferential PSLs. The green arrows indicate the positions of the boundaries between the PbS and CdS domains.

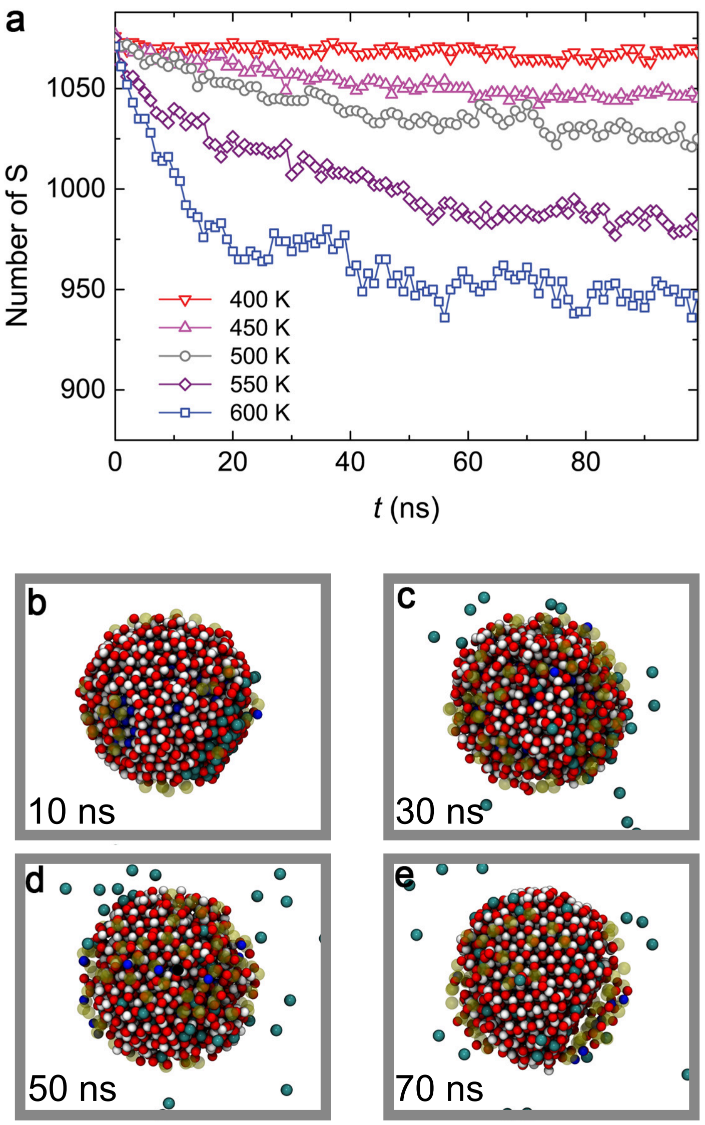

Supplementary Figure 5: **The dissolution of anions in CE. a** Time evolution of the number of S ions remaining in the NC at different temperatures. **b-e** Typical MD snapshots of a PbS NC during CE at 600 K. The white, blue, red, and yellow spheres represent S, Pb, Cd, and PSLs, respectively. Several S ions are marked in cyan in **b**. These S ions dissolve in the surrounding solution and participate in the NC reconstruction in (**c-e**).

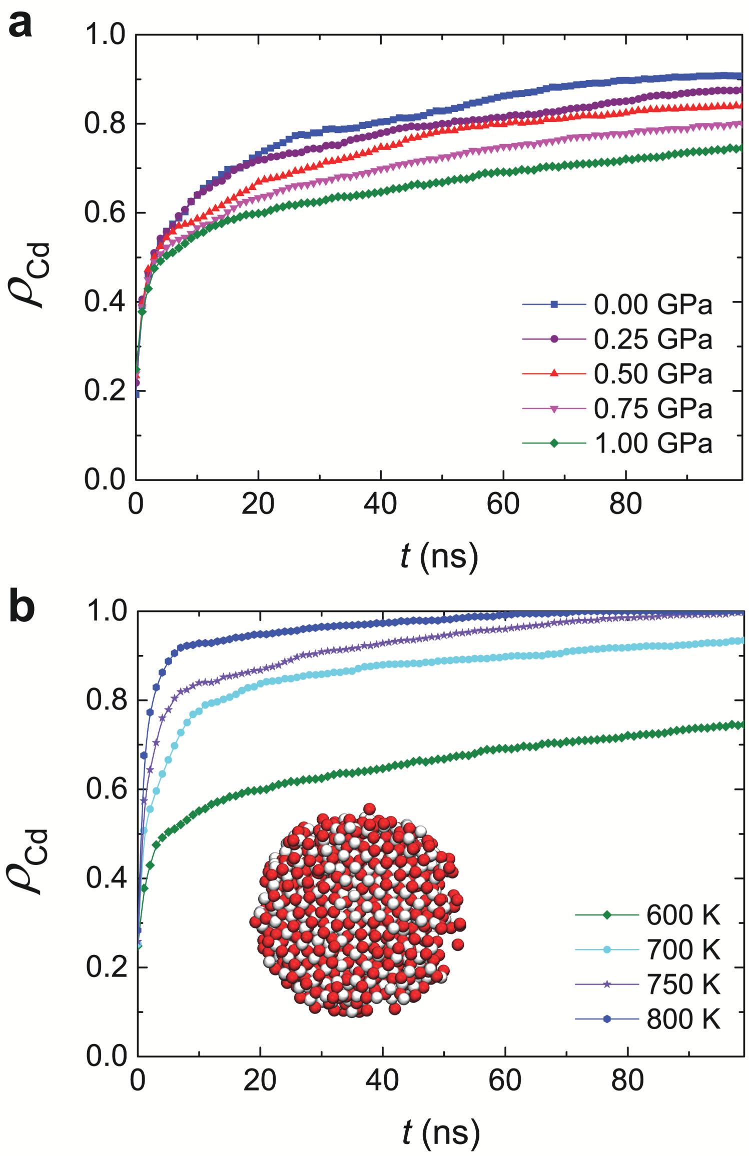

Supplementary Figure 6: **Pb→Cd CE at high-temperature-high-pressure conditions.** **a** Time evolution of the exchange ratio ρ_{Cd} at 600 K for different hydrostatic pressures in the range of 0.00–1.00 GPa. **b** Time evolution of exchange ratio ρ_{Cd} at temperatures of 600, 700, 750, and 800 K and at a pressure of 1.00 GPa. Simulations were performed with Pb-preferential PSLs for 100 ns. The inset in **b** shows the final configuration of an exchanged NC at 800 K and 1.00 GPa, in a CdS-[001] projection. The white and red spheres represent S and Cd ions, respectively.

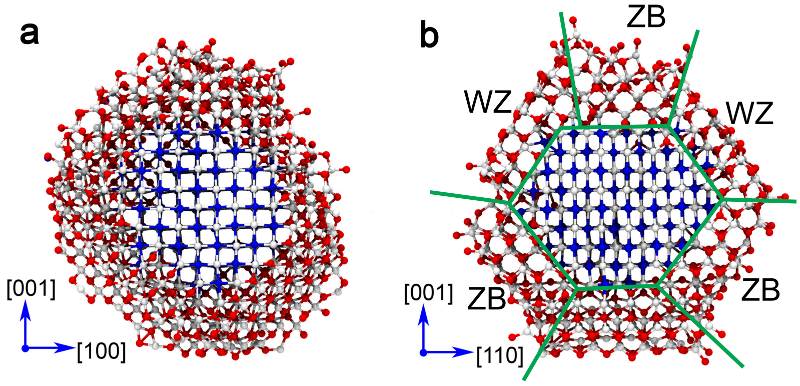

Supplementary Figure 7: **Final configuration of a CdS/PbS core/shell HNC**. The configuration is obtained from a MD simulation performed at 550 K with Pb-preferred PSLs for 300 ns. The core/shell HNC is shown along PbS-[010] in **a** and along PbS-[011] direction in **b**. The green lines in **b** indicate the boundaries between the WZ-CdS, ZB-CdS, and RS-PbS domains. The structures shown in this figure are represented by sticks and spheres with white, blue, and red colours for S, Pb, and Cd ions, respectively.

Supplementary Table 1: **Parameters of the Buckingham potentials in the coarse-grained PSL model.** The non-preferential, Pb-preferred, and Cd-preferred PSL models are constructed using different combinations of the cation-PSL potentials with different values of the parameter C.

	A (eV)	B (Å)	C (eV·Å^6)
**non-preferential PSL**			
Cd-PSL	1000	0.4	0
Pb-PSL	1000	0.4	0
S-PSL	15000	0.4	0
PSL-PSL	0	0.4	0
**Pb-preferred PSL**			
Cd-PSL	1000	0.4	0
Pb-PSL	1000	0.4	200
S-PSL	15000	0.4	0
PSL-PSL	0	0.4	0
**Cd-preferred PSL**			
Cd-PSL	1000	0.4	200
Pb-PSL	1000	0.4	0
S-PSL	15000	0.4	0
PSL-PSL	0	0.4	0

Supplementary Table 2: **Statistics of the Pb jumps.** Statistics were extracted from five independent 200 ns MD simulations (marked as MD #1-5) for a PbS NC with non-preferential PSLs at 550 K. n_{intf}, n_{shell}, and n_{kick} are the numbers of identified Pb jumps at the PbS/CdS interface, in the CdS shell region, and at the interface with at least one nearby Cd interstitial, respectively.

	MD #1	MD #2	MD #3	MD #4	MD #5	total	mean (s.e.m.)
n_{intf}	1188	1258	1731	1585	1215	6977	
n_{shell}	498	684	650	1128	756	3716	
n_{kick}	563	516	848	715	661	3303	
$n_{\mathrm{kick}}/n_{\mathrm{intf}}$	0.474	0.410	0.490	0.451	0.544		0.474 (0.022)

Supplementary Table 3: **Averaged distances between migrating Pb ions and their four nearest anions and cations.** $\bar{r_a}$ and $\bar{r_c}$ are the averaged distances between the migrating Pb ions and their four nearest anions and cations, respectively, after the jump. "Intf" and "Shell" denote the Pb ion jumps taking place at the PbS/CdS interface and in the CdS shell region, respectively. "octa" and "tetra" denote the Pb jumps into octahedral and tetrahedral cation sites, respectively. All distances are in Å.

	Intf-octa	Intf-tetra	Shell-octa	Shell-tetra
$\bar{r_a}$	3.02	3.04	3.07	3.10
$\bar{r_c}$	3.22	3.42	3.31	3.48
$\bar{r_c}/\bar{r_a}$	1.07	1.13	1.08	1.12

Supplementary Discussion

We introduced a pseudoligand (PSL) model to understand the effects of different cation-ligands interactions on the CE process. This coarse-grained PSL model was not developed for exactly reproducing physical or chemical properties of any specific ligand. The solution of cations and PSLs is essentially an ionic liquid. The parameters used in the PSL model were developed and determined by the following criteria:

1. The cation-PSL interactions should be weaker than the cation-S interactions.
2. The size of a PSL should be sufficiently large and the repulsion between S ions and the PSL should be strong enough to prevent PSLs from penetrating into the NC.
3. The cation-PSL systems should be in a liquid phase at the reaction temperatures (400∼800 K) and at zero pressure.
4. MD simulations using the PSL model should be able to reproduce CE showing a behaviour similar to experimental CE.
5. Using this model, both the Pb→Cd and the reversed Cd→Pb CE processes should proceed at moderate rates so that a clear core/shell structure can be observed in a 100 ns MD simulation at 550 K (*i.e.*, a model such that CE takes place in a timescale accessible by MD simulations).

Supplementary Fig. 2 shows the radial distribution function $g(r)$ for cations and PSLs in solutions at 500 K. Since CE takes place in the NC, we expect that the liquid structure has a small effect on the mechanism of the CE process. However, one limitation of the PSL model is that it cannot model chemical reactions which may play a role in the CE at the NC surface.[1]

Although the coarse-grained PSL model is able to effectively reproduce the CE process in colloidal NCs, it differs from solutions used in CE experiments:

1. The concentration of cations in the solution in our simulations ($\sim$ 7 M) is higher than that in experiments[2–4] (0.1∼0.3 M). Additionally, in experiments, the concentration of metal-ligands close to the NC surface is lower than that in the bulk solution. Our simulation box is too small to reproduce the concentration profiles. These factors may attribute to a relatively high concentration of the guest cations at the NC surface in our simulations thereby accelerating the CE process.
2. Solvent molecules are not explicitly considered in our simulations. To integrate the effects of solvent molecules, we assigned a relatively large mass for the PSLs. The mass of the PSLs was set at 500 grams/mole in our simulations. We also performed test simulations for the Pb→Cd CE using PSLs with masses of 250 and 1000 grams/mole. Those simulations show nearly identical results.
3. The size of PSLs is smaller than typical ligands used in CE experiments (*e.g.*, oleate acid), thus a strong PSL-S interaction is needed to prevent PSL exchange with S ions. The current parameter set for the PSL model is able to prevent the PSLs penetrating into the NC. Test simulations with a smaller value of parameter A (10000 eV) for the S-PSL interactions (less repulsion between S ions and PSLs) show similar results for the CE process without any PSL penetrating into the NC.

4. The PSL model with rigid point charges is not able to reproduce chemical reactions in the solution.

Improving the coarse-grained PSL model to investigate the effects of ligands/solvents on the CE process is of great interest and an important topic for future investigations.

Supplementary Methods

Coarse-Grained Pseudoligand Model

In the pseudoligand (PSL) model, the functional form of the PSL-PSL and PSL-ion interatomic interaction potentials in the coarse-grained PSL model is identical to that for the PbS-CdS systems:[5]

$$u_{ij}(r_{ij}) = \frac{q_i q_j}{r_{ij}} + Ae^{-r_{ij}/B} - \frac{C}{r_{ij}^6} \tag{1}$$

The first term describes the Coulombic interactions whereas the second and the third terms are the Buckingham potential. The partial charges of PSLs were set to -0.8 e. The parameters of the Buckingham potential are listed in Supplementary Table 1. To control the strength of the PSL-cation interactions, we varied the parameter C for PSL-cation interactions. The parameter C of the PSL-cation interatomic potentials can be chosen as 0 or 200 eV·Å^6. When keeping other parameters unchanged, a PSL-cation interaction potential with C = 200 eV·Å^6 has a lower minimum energy compared to that with C = 0 eV·Å^6 (see Supplementary Fig. 1). The non-preferential, Pb-preferred, and Cd-preferred PSL models can be constructed using different combinations of the PSL-cation potentials with different values of parameter C (see Supplementary Table 1).

Reverse Cd→Pb CE in Colloidal ZB-CdS NCs

The construction of colloidal ZB-CdS NCs is similar to that of RS-PbS NC systems described in the Methods section of the main text. A spherical CdS NC with a diameter of 4.7 nm containing 1057 {Cd–S} pairs was cut out from a ZB-CdS matrix with a lattice parameter of 6.00 Å. 4228 {Pb–PSL} pairs were randomly positioned inside a cubic simulation box of a size of 10 nm. MD simulations were performed for the colloidal ZB-CdS NC for 100 ns at 550 K with non-preferential, Pb-preferred, and Cd-preferred PSLs. Details of the simulations are identical to those for the RS-PbS NC systems described in the Methods section of the main text.

Statistical Analysis for the Kinetic Mechanism

To study the kinetic mechanism of CE, statistics were extracted from five independent MD simulations. Each simulation run was performed for the PbS NC at 550 K using non-preferential PSLs for 200 ns, and the atomic trajectories were saved every 2 ps. We used the following criteria to identify Pb jumps in our simulations:

1. The Pb ion moved in a time interval of 2 ps over a distance larger than 2.0 Å.
2. The distance between the Pb ion and the centre of the NC is less than 20.0 Å.
3. The Pb ion jumps away from the centre of the NC.
4. The number of nearby Pb ions (<4.5 Å) around the jumping Pb ion is larger than 2.
5. There is no nearby Pb ion around the jumping Pb ion.

If the motion of a Pb ion meets criteria 1, 2, 3, and 4, this motion is identified as a Pb jump at the PbS/CdS interface. If the motion of a Pb ion meets criteria 1, 2, and 5, this motion is identified as a Pb jump in the CdS shell region.

To study these identified Pb jumps, the configurations before (pre-jump) and after (post-jump) each Pb jump were extracted from the simulations for further analysis.. The time interval between two snapshots is 2 ps. We analysed the atomistic environments (*i.e.*, the distribution of cations and anions) around the pre-jump and post-jump locations of each jumping Pb ion. To validate the "kick-out" mechanism, we searched for exchanged Cd ions around the pre-jump locations of the jumping Pb ions in the pre-jump configurations. An exchanged Cd ion was identified if the distance between the Cd ion and the jumping Pb is less than 3.6 Å and the Cd ion is in the PbS core region. To distinguish whether Pb ions jump into tetrahedral or octahedral cation sites, we counted the number of the nearest anions around the post-jump locations of the jumping Pb in the pre-jump configurations. A nearest anion is defined as the distance between the post-jump location and the anion is less than 4.1 Å. To distinguish whether Pb ions migrate through interstitial sites or through vacancies, we calculated the averaged distances between jumping Pb ions and their four nearest cations ($\bar{r_c}$) and anions ($\bar{r_a}$) in the post-jump configurations. The results are shown in Supplementary Tables 2 and 3 and in Fig. 5d of the main text.

Simulations of CE at High-Temperature High-Pressure Conditions

MD simulations for PbS NCs in Cd-PLS solutions at high-pressure-high-temperature conditions were performed as well. The simulation details are similar to those at zero pressure (see Method Section of the main text). Different hydrostatic pressures were applied to the system using the NPT ensemble. To investigate the effect of pressure on Pb→Cd CE, five independent simulations were performed for a PbS colloidal NC at a temperature of 600 K but with different pressures. The pressure ranged from 0 to 1.00 GPa with an increasing step of 0.25 GPa. In addition, at a fixed pressure of 1.0 GPa, MD simulations were performed for the same PbS colloidal NC system at 700, 750, and 800 K. Supplementary Figure. 6a shows the exchange ratio as a function of simulation time for different pressures at 600 K. Supplementary Figure. 6b shows the exchange ratios as a function of simulation time at 1.00 GPa for different temperatures. Clearly, at elevated pressures, the exchange ratio of Pb→Cd CE is slightly decreased. High-pressure conditions are potentially able to protect PbS NC from being damaged by high temperatures, thus enabling very fast CE at high temperatures (Supplementary Fig. 6b).

Supplementary Reference

[1] Zherebetskyy, D.; Scheele, M.; Zhang, Y.; Bronstein, N.; Thompson, C.; Britt, D.; Salmeron, M.; Alivisatos, P.; Wang, L.-W. Hydroxylation of the Surface of PbS Nanocrystals Passivated with Oleic Acid *Science* **2014**, *344*, 1380–1384.

[2] Pietryga, J. M.; Werder, D. J.; Williams, D. J.; Casson, J. L.; Schaller, R. D.; Klimov, V. I.; Hollingsworth, J. A. Utilizing the Lability of Lead Selenide to Produce Heterostructured Nanocrystals with Bright, Stable Infrared Emission. *J. Am. Chem. Soc* **2008**, *130*, 4879–4885.

[3] Zhao, H.; Chaker, M.; Wu, N.; Ma, D. Towards Controlled Synthesis and Better Understanding of Highly Luminescent PbS/CdS Core/Shell Quantum Dots. *J. Mater. Chem.* **2011**, *21*, 8898–8904.

[4] Justo, Y.; Sagar, L. K.; Flamee, S.; Zhao, Q.; Vantomme, A.; Hens, Z. Less Is More. Cation Exchange and the Chemistry of the Nanocrystal Surface. *ACS Nano* **2014**, *8*, 7948–7957.

[5] Fan, Z.; Koster, R. S.; Wang, S.; Fang, C.; Yalcin, A. O.; Tichelaar, F. D.; Zandbergen, H. W.; van Huis, M. A.; Vlugt, T. J. H. From Sphere to Multipod: Thermally Induced Transitions of CdSe Nanocrystals Studied by Molecular Dynamics Simulations. *J. Chem. Phys.* **2014**, *141*, 244503.
